# Role of brain renin–angiotensin system in depression: A new perspective

**DOI:** 10.1111/cns.14525

**Published:** 2023-11-12

**Authors:** Naif H. Ali, Hayder M. Al‐Kuraishy, Ali I. Al‐Gareeb, Ali K. Albuhadily, Rabab S. Hamad, Athanasios Alexiou, Marios Papadakis, Hebatallah M. Saad, Gaber El‐Saber Batiha

**Affiliations:** ^1^ Department of Internal Medicine Medical College Najran University Najran KSA; ^2^ Department of Clinical Pharmacology and Medicine, College of Medicine Mustansiriyah University Baghdad Iraq; ^3^ Biological Sciences Department College of Science, King Faisal University Al Ahsa Saudi Arabia; ^4^ Central Laboratory Theodor Bilharz Research Institute Giza Egypt; ^5^ University Centre for Research & Development, Chandigarh University Mohali Punjab India; ^6^ Department of Science and Engineering Novel Global Community Educational Foundation Hebersham New South Wales Australia; ^7^ AFNP Med Wien Austria; ^8^ Department of Surgery II University Hospital Witten‐Herdecke, University of Witten‐Herdecke Wuppertal Germany; ^9^ Department of Pathology, Faculty of Veterinary Medicine Matrouh University Matrouh Egypt; ^10^ Department of Pharmacology and Therapeutics, Faculty of Veterinary Medicine Damanhour University Damanhour AlBeheira Egypt

**Keywords:** angiotensin, angiotensin receptor blockers, angiotensin‐converting enzyme inhibitors, depression, renin–angiotensin system

## Abstract

Depression is a mood disorder characterized by abnormal thoughts. The pathophysiology of depression is related to the deficiency of serotonin (5HT), which is derived from tryptophan (Trp). Mitochondrial dysfunction, oxidative stress, and neuroinflammation are involved in the pathogenesis of depression. Notably, the renin–angiotensin system (RAS) is involved in the pathogenesis of depression, and different findings revealed that angiotensin‐converting enzyme inhibitors (ACEIs) and angiotensin receptor blockers (ARBs) may be effective in depression. However, the underlying mechanism for the role of dysregulated brain RAS‐induced depression remains speculative. Therefore, this review aimed to revise the conceivable role of ACEIs and ARBs and how these agents ameliorate the pathophysiology of depression. Dysregulation of brain RAS triggers the development and progression of depression through the reduction of brain 5HT and expression of brain‐derived neurotrophic factor (BDNF) and the induction of mitochondrial dysfunction, oxidative stress, and neuroinflammation. Therefore, inhibition of central classical RAS by ARBS and ACEIs and activation of non‐classical RAS prevent the development of depression by regulating 5HT, BDNF, mitochondrial dysfunction, oxidative stress, and neuroinflammation.

## Graphical abstract

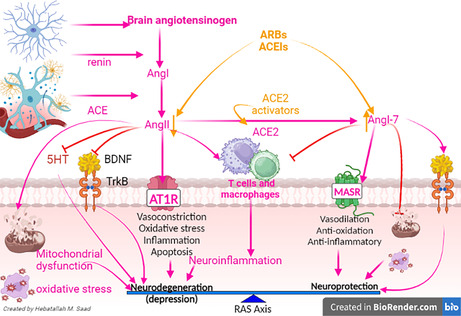

## 1 INTRODUCTION

Depression is a mental state of low mood and aversion to activity characterized by abnormal mood and thoughts.^1^ Depression is associated with low extraversion, and people who have high levels of neuroticism are more likely to experience depressive symptoms and to receive a diagnosis of a depressive disorder.^1^ Depression affects 3.5% of the general population.^2^ Depression involves a triad of symptoms, including depressed mood, fatigue, and anhedonia, as well as other symptoms such as sleep disorders and autonomic dysfunction‐mediated gastrointestinal disturbances.^3^ Depression is more common in women than men and can happen at any age, leading to disruption of life. If untreated, it can be fatal.^4^, ^5^ In relation to the etiology, depression is classified as endogenous depression due to genetic factors or reactive depression due to external stimuli.^6^ The risk factors for development are multifactorial, including stressful life events,^7^ borderline personality disorders,^8^ chronic use of sedatives and hypnotics, and adverse effects from long‐term use of β‐blockers, antipsychotic drugs, isotretinoin, and antimigraine medications.^9^, ^10^ Moreover, psychiatric disorders such as bipolar disorders, major depressive disorder (MDD) episodes, and seasonally affected disorders increase the risk of the development of depression.^11^ Additionally non‐psychiatric disorders, including nutritional deficiencies,^12^ infectious diseases,^13^ Addison disease,^14^ hypothyroidism,^15^ Cushing disease,^16^ hyperparathyroidism,^17^ Parkinson's disease (PD)^18^ and multiple sclerosis,^19^ trigger the incidence of depression.

There is evidence for a link between inflammation and depression. Inflammatory processes can be triggered by negative cognitions or their consequences, such as stress, violence, or deprivation. Thus, negative cognitions can cause inflammation that can, in turn, lead to depression. In addition, there is increasing evidence that inflammation can cause depression because of the increase of cytokines, setting the brain into a “sickness mode”.^13^ Classical symptoms of being physically sick, such as lethargy, show a large overlap in behaviors that characterize depression. Levels of cytokines tend to increase sharply during the depressive episodes of people with bipolar disorder and drop off during remission. Inflammations that lead to serious depression could be caused by common infections caused by viruses, bacteria, or even parasites.^13^

Depressive mood might be a symptom of other mood disorders, including dysthymia and MDD.^1^, ^2^ The depression term was derived from the Latin word deprimere, which means to press down. This term was used previously by English authors Richard Baker and Samuel Johnson in 1665 and 1753, respectively.^4^ Changes in brain neurotransmitter theory were postulated in the 1950s when anti‐tuberculosis medications isoniazid and reserpine were used, causing depressive symptoms.^5^ In the late 1960s and early 1970s, depression was categorized as a unipolar and bipolar mood disorders. Unipolar and bipolar mood disorders were primarily described by German psychiatrist Karl Kleist.^3^

**FIGURE 1:**
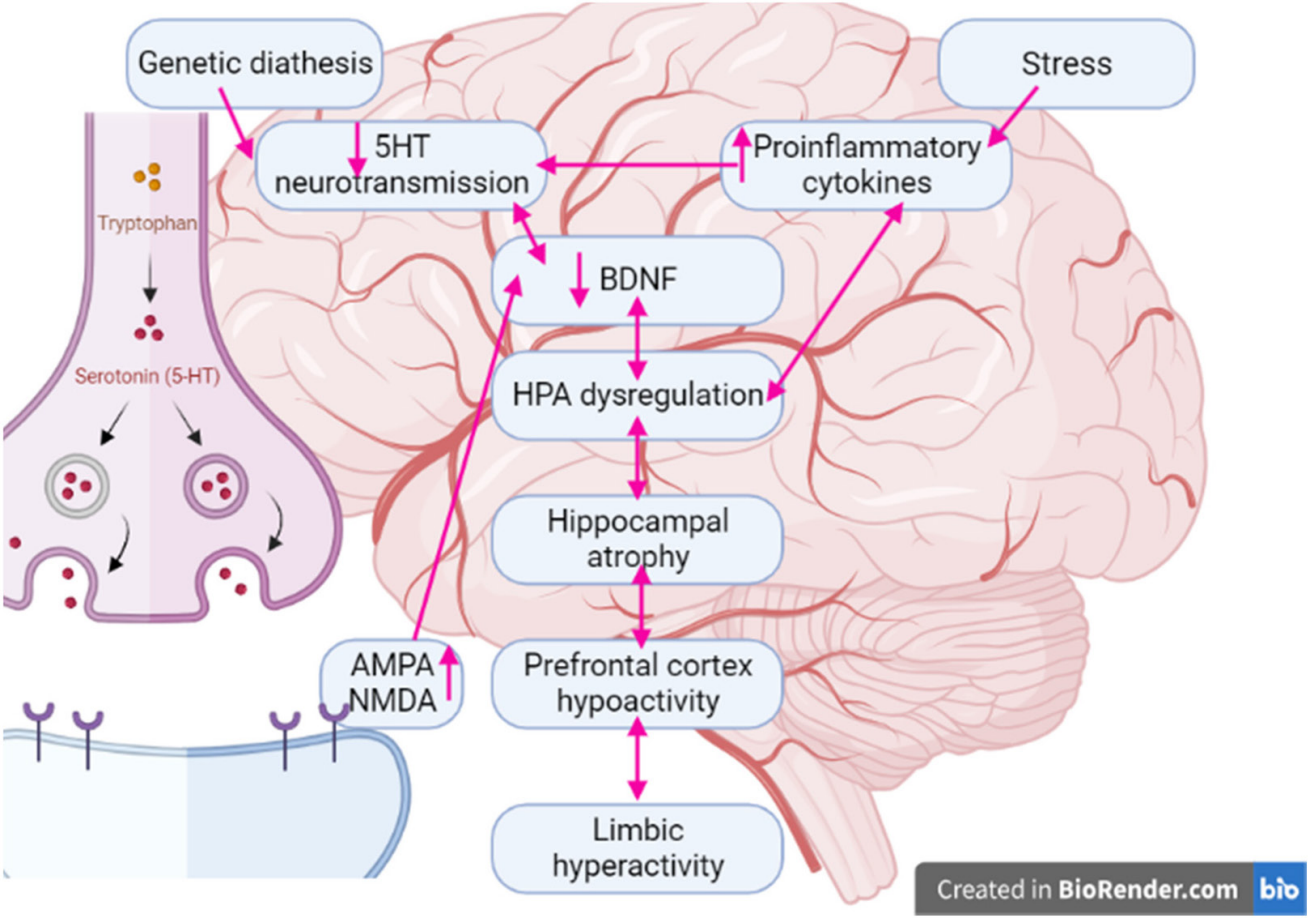
Pathophysiology of depression.

The pathophysiology of depression is related to the deficiency of serotonin (5HT), which is derived from tryptophan (Trp). 5HT is released into the synaptic cleft to act on the post‐synaptic 5HT and on the presynaptic 5HT1A, which acts as an autoreceptor.^20^ Consequently, the increase of 5HT1A autoreceptors and the reduction of 5HT1A heteroreceptors are intricate in the pathogenesis of depression by reducing glutamate.^20^, ^21^ This effect induces upregulation of *N*‐methyl‐d‐aspartate (NMDA) receptors and upregulation of amino‐methyl propionic acid (AMPA) receptors, leading to the reduction of brain‐derived neurotrophic factor (BDNF) and inhibition of neuronal plasticity^20^, ^21^ (Figure 1).

Alongside the well‐known deficiency in 5HT neurotransmission as a pathophysiological correlate of depression, different evidence points to a pivotal role of increased glutamate receptor activation as well.^22^ An immune activation, including increased production of pro‐inflammatory cytokines such as interleukin‐2 (IL‐2), interferon‐gamma (INF‐Y), and tumor necrosis factor‐alpha (TNF‐α), activate the tryptophan and 5HT‐degrading enzyme indoleamine 2,3‐dioxygenase (IDO).^23^ Depressive states during inflammatory somatic disorders are also associated with increased pro‐inflammatory cytokines and increased consumption of tryptophan via activation of IDO.^22^, ^23^ Enhanced consumption of 5HT and its precursor tryptophan through IDO activation could well explain the reduced availability of 5HT neurotransmission in depression. An increased activation of IDO and its subsequent enzyme kynurenine monooxygenase by pro‐inflammatory cytokines, moreover, leads to an enhanced production of quinolinic acid, a strong agonist of the NMDA. In inflammatory states, IDO is mainly activated in microglial cells, which preferentially metabolize tryptophan to the NMDA receptor agonist quinolinic acid, whereas astrocytes counteract this metabolism due to the lack of an enzyme for this metabolism, which has been observed to be reduced in depression.^22^, ^23^ Therefore, the type 1/type 2 immune response imbalance, associated with an astrocyte/microglia imbalance, leads to 5HT deficiency and glutamatergic overproduction.^24^ Astrocytes are further strongly involved in the reuptake and metabolic conversion of glutamate. The reduced number of astrocytes could contribute to both a diminished counterregulation of IDO activity in microglia and an altered glutamatergic neurotransmission.^24^ Further search for antidepressant agents should take into account anti‐inflammatory drugs; for example, cyclooxygenase‐2 (COX2) inhibitors might exert antidepressant effects by acting on 5HT deficiency, glutamatergic hyperfunction, and the antagonizing neurotoxic effects of quinolinic acid.^25^

Despite these findings, the pathophysiology of depression is still elusive, and existing treatments that mainly focus on monoamine alterations are only partially effective. In addition, the effectiveness of antidepressant agents is only 30%.^26^ Therefore, focusing on mitochondrial dysfunction, oxidative stress, and neuroinflammation could be a novel avenue in the management of depression. These cellular mechanisms are mainly regulated by the renin–angiotensin system (RAS).^27^ Notably, RAS is involved in the pathogenesis of depression,^26^ and different studies revealed that angiotensin‐converting enzyme inhibitors (ACEIs) and angiotensin receptor blockers (ARBs) may be effective in depression.^28^ Thus, this review aimed to revise the possible role of ACEIs and ARBs and how these agents ameliorate the pathophysiology of depression.

## 2 A BRIEF OVERVIEW OF RAS

RAS was primarily regarded as a humoral system involved in regulating water and sodium homeostasis and controlling blood pressure.^29^ Of note, RAS is phylogenetically one of the important hormone systems intricate in human evolution.^29^ RAS consists of various peptides and enzymes. Angiotensinogen from the liver is regarded as the main precursor for the formation of angiotensin I (AngI) by the effect of renin, which is released from the kidney.^30^ AngI is converted by the effect of ACE to AngII, which acts on AngII receptors AT1 and AT2. AT1 induces vasoconstriction and pro‐inflammatory effects, though AT2 mediates the effect of vasodilation and anti‐inflammatory effects of AngII. Besides, ACE2 promotes the conversion of AngI to Ang1‐9 and AngII to Ang1‐7, which, via activation of the MAS receptor, induces an anti‐inflammatory effect.^31^

Furthermore, in addition to its systemic effects, RAS can produce local and paracrine effects.^32^ Both systemic and local RAS act mutually in different tissues.^32^ Importantly, the upregulation of AngII and activation of AT1 trigger the development of oxidative stress and inflammation by activating NADPH oxidase and inducing mitochondrial dysfunction.^33^ Notably, NADPH oxidase is upregulated during the aging process and aging‐related disorders such as diabetes, hypertension, and atherosclerosis. However, stimulation of AT2 by AngII leads to an opposing effect characterized by inhibition of inflammation and oxidative stress mediated by AT1.^34^ AT2 inhibits the release of pro‐inflammatory cytokines, chemokines, and expression of adhesion molecules.^34^ Therefore, RAS has a dual inflammatory effect via the AngII/AT1 axis and an anti‐inflammatory effect via the AngII/AT2 axis.

## 3 BRAIN RAS

The potential role of RAS on the brain was attributed to its local effect in the area engaged with the regulation of water/sodium homeostasis and blood pressure that lacks the blood–brain barrier (BBB).^35^ Later on, all components of RAS were recognized in various brain regions, and the AngII level was higher in the brain than in the peripheral circulation.^36^ Interestingly, angiotensinogen, pro‐renin, and renin cannot cross the BBB.^37^ However, pro‐renin has been detected in the brain, but its origin has not been identified.^38^ Neuronal pro‐renin, via activation of the pro‐renin receptor, promotes cleavage of angiotensinogen and activation of RAS.^38^ Surprisingly, neuronal pro‐renin/renin may be involved in many neurological functions not related to RAS, like neurogenic hypertension.^38^, ^39^

Besides, brain angiotensinogen is not correlated with the circulating one.^40^ It has been shown that 90% of brain angiotensinogen is mainly produced by astrocytes and little from neurons and glial cells.^41^ In addition, components of RAS as well as pro‐renin are also expressed in the basal ganglion, mainly in the nigrostriatal pathway.^42^ AT1, AT2, and pro‐renin receptors are highly expressed in glial cells, suggesting the role of RAS in inflammatory and oxidative stress in the brain.^43^ In particular, RAS components are expressed in intracellular parts of the neurons.^32^ Of note, brain AngI is an inactive peptide and is found at a low level.^44^ However, AngII is highly abundant in the brain and intricately involved in different physiological and neuropathological alterations.^45^ AngII is further converted to AngIII by the aminopeptidase enzyme; AngIII activates AT1 and AT2 receptors as well as non‐Ang receptors.^46^ AngIII is further converted to AngIV by the aminopeptidase enzyme AngIV, which acts on the AT4 receptor and has a neuroprotective effect.^47^

Furthermore, ACE, which is the primary enzyme in RAS, is expressed in the astrocytes, choroid plexus, and cerebral vascular endothelium.^48^ Brain ACE is mainly present in regions lacking BBB, though it is also expressed in the neurons and glial cells.^48^ However, ACE2 is chiefly expressed in brain regions intricately involved in the regulation of blood pressure.^49^ ACE2 promotes the expression of neuroprotective Ang1‐7 and Ang1‐9, particularly the ACE2/Ang1‐7/MASR axis, which has anti‐inflammatory and antioxidant effects.^50^ In the brain, the AT2 receptor is less expressed than the AT1 receptor and restricted to some brain regions.^51^ However, binding of AngII to the AT1 receptor induces oxidative stress and inflammation by activating mitogen‐activated protein kinase (MAPK) and the JNK signaling pathway.^52^ These changes may lead to neurotoxicity by activating NMDA receptors and inducing BBB impairment.^52^ In addition, the AngII/AT1 receptor complex is translocated into the nucleus, triggering the activation of RAS components.^53^ Specifically, mitochondrial AT1 via activation of NADPH oxidase and the respiratory chain promotes the generation of reactive oxygen species (ROS) and the development of oxidative stress.^54^ NADPH oxidase is highly expressed in glial cells and neurons.^55^ However mitochondrial AT2 produces the opposite effect, as the AngII/AT2 receptor complex enhances the expression of nuclear sirtuin 1 (SIRT1), which has a neuroprotective effect.^56^, ^57^

It has been shown that over‐activation of brain RAS is associated with cognitive impairment and the development of neurodegenerative diseases.^58^ ACE over‐activity in the dopaminergic neurons of the substantia nigra increases vulnerability to damage to these neurons and the development of PD.^59^, ^60^ Therefore, ACEIs and ARBs are effective against motor dysfunction and cognitive impairment in PD.^56^, ^61^ Similarly, ACEIs and ARBs are effective against Alzheimer's disease (AD) neuropathology by inhibiting inflammation and oxidative stress‐induced neuronal injury.^62^, ^63^ Moreover, over‐activity of RAS is correlated with disease severity in different neurodegenerative disorders, including dementia, multiple sclerosis, amyotrophic lateral sclerosis, Huntington's disease, stroke, and traumatic brain injury.^64^, ^65^

Furthermore, dysregulation of brain RAS is also implicated in different mental and psychiatric disorders, such as bipolar disorder.^66^ Bipolar disorder, previously known as manic depression, is a mental disorder characterized by periods of depression and periods of abnormally elevated mood that each last from days to weeks. It has been observed that brain RAS activity is augmented and correlated with the severity of bipolar disorder.^66^ Recent studies have linked RAS not only with neuro‐immunological processes but also with psychiatric conditions like mood and anxiety disorders. Anxiety disorders represent a heterogeneous group of illnesses that are characterized by excessive fear and anxiety, hypervigilance, and related behavioral disturbances. Anxiety is one of the most common comorbid disorders with MDD. A large psychiatric cohort study has reported that depression preceded anxiety in 18% of such comorbid cases, while in 57% of the cases, anxiety preceded depression.^67^ It has been shown that anxiety associated with depression often leads to reduced responses and decreased compliance with depression pharmacotherapy.^68^ Of note, increased Ang II level is linked with depression, anxiety, hyperactivity of the HPA axis, and stress.^69^ Moreover, hyperactivation of AT_1_ receptors is associated with promoting anxiety‐like behaviors in the brain. Deletion of AT_1_ receptors from the paraventricular nucleus (PVN) reduced anxiety‐like behaviors in mice.^70^

Clinical studies show associations between RAS and mood disorders. Animal studies on mood disorder models, either depression or mania, were focused on the reversal of behavioral and/or cognitive symptoms through the inhibition of RAS components like the ACE, AT1, or Mas receptors.^71^ ACE polymorphisms are linked to mood disorders and suicidal behavior. Hypertension was associated with neurocognitive deficits in mood disorders, which were reversed with RAS inhibition. Low levels of RAS components and mood symptoms improved with ACE inhibitors or AT1 blockers were also observed in mood disorders.^71^ Epidemiological, genetic, and biochemical findings hypothesize the possible relationship between RAS and suicide, as the RAS is involved in the neurobiology of suicide,^72^ although the exact mechanisms underlying this involvement are still unidentified. On the other hand, some epidemiological studies have raised alarms about conceivable associations between the use of medications targeting the RAS and an increased risk of suicide. In particular, the association between the use of ARBs and suicide has been discussed not only in the specialized literature but also in the mass media due to the potential implications and the widespread use of these medications in the treatment of hypertension and other cardiovascular conditions.^73^, ^74^ Moreover, the central RAS affects inflammation, glutamate, dopamine, gamma‐aminobutyric acid (GABA), and peroxisome proliferator‐activated receptor (PPAR)‐γ, all of which are associated with schizophrenia etiology. In addition, it has been demonstrated the therapeutic potential of RAS modulators, especially ARBs, as adjunctive therapy to the currently available antipsychotic medications for schizophrenia treatment.^75^ With a greater understanding of how RAS inhibition directly modulates neurotransmitter balance in the brain, it is possible that compounds with RAS‐inhibiting properties could be used to optimize physiological levels of glutamate, dopamine, and GABA and the balance among the three neurotransmitters, analogously to how antipsychotic medications mediate the dopaminergic pathways. It can be hoped that a novel approach based on this concept, such as adjunctive telmisartan therapy, may offer practical interventional strategies to address currently unmet therapeutic needs in patients with schizophrenia, especially those with treatment‐resistant schizophrenia. A case–control study that involved 25 patients with schizophrenia and 20 healthy controls revealed that plasma levels of ACE were reduced in schizophrenic patients compared to healthy controls; no significant differences were found regarding ACE2, Ang‐1–7, and Ang II levels. There were no associations between the measured molecules and clinical parameters,^76^ suggesting that the RAS is involved in the pathophysiology of schizophrenia. These findings indicated that brain RAS is implicated in the pathogenesis of mental and psychiatric disorders.

**FIGURE 2:**
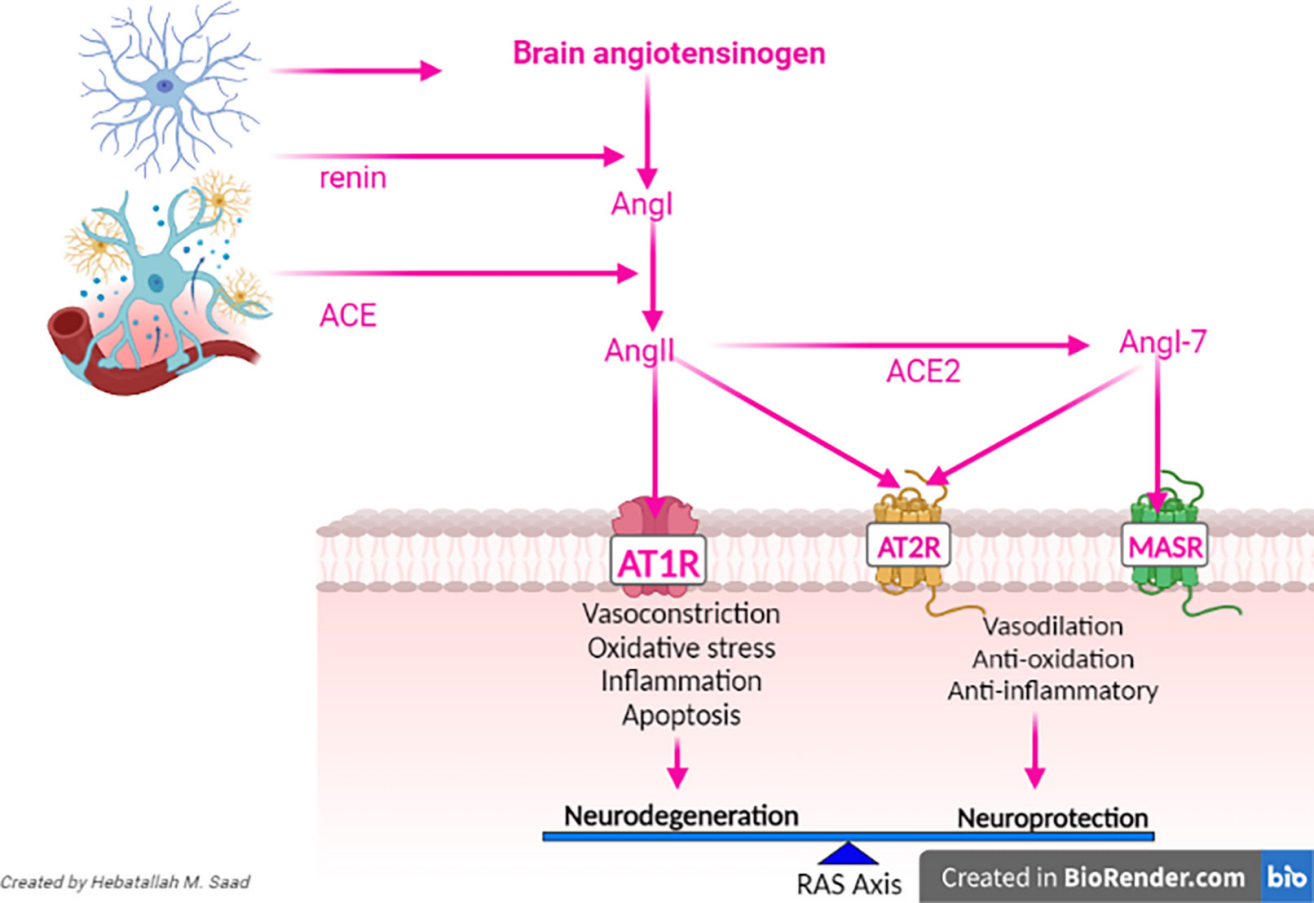
Brian renin–angiotensin system.

Collectively, brain RAS has two arms: the harmful one is mediated by the AngII/AT1 receptor, and the protective arm is mediated by the AngII/AT2 receptor and the ACE2/Ang1‐7/MASR axis (Figure 2).

## 4 HYPERTENSION AND DEPRESSION

It has been shown that depression increases the risk of hypertension, and the high prevalence of depression among hypertensive patients suggests a potential association between them.^77^ One possible link between depression and hypertension is sympathetic over‐activity.^77^ Besides, the use of antidepressant agents may interfere with blood pressure control by inducing circadian alteration in the blood pressure.^78^ A cross‐sectional, population‐based study that involved 5000 subjects with hypertension showed that subjects with unaware and uncontrolled hypertension were inversely correlated with depression risk, though controlled hypertension was positive for depression risk.^78^ This finding suggests that depression risk in hypertensive patients is related to hypertensive treatments, which may affect mood and cognitive functions. Conversely, low adherence to antihypertensive drugs and uncontrolled hypertension increase the risk of depression.^78^ However, a large population‐based study revealed that 16.7% of hypertensive patients have 12‐month depressive and anxiety disorders. After adjusting for confounding factors, there was no association between hypertension and 12‐month depressive disorders.^79^ These observations proposed a controversial verdict regarding the association between depression and hypertension. A systematic review and meta‐analysis included 41 clinical studies observed that 26.8% of hypertensive patients have depression.^80^ This association seems to be similar with other chronic diseases, as depression risk is 20.3% in chronic kidney disease and 19.3% in patients with heart failure.^81^, ^82^ Furthermore, depressive symptoms are common in hypertensive patients, even without comorbidities.^83^ Remarkably, depression augments the risk of the development of hypertension.^84^ A meta‐analysis illustrated that depression is regarded as an independent risk factor for the development and progression of hypertension.^84^ Depression increases hypertension risk by inducing dysregulation of Ca^2+^/cylic adenosine monophosphate (cAMP) signaling, which is intricate in the pathogenesis of hypertension. Furthermore, oxidative stress, inflammation, mitochondrial dysfunction, and endoplasmic reticulum stress, which are common in both depression and hypertension, could be the possible links between them.^85^, ^86^ Therefore, early management of depression and hypertension prevents the development and progression of a pathogenic link between them. One of the most commonly used antihypertensive agents are ARBs and ACEIs, which modulate peripheral and central RAS. Thus, brain RAS is implicated in the pathogenesis of depression, and modulation of this system may ameliorate the development and progression of depression.

## 5 BRIAN RAS AND DEPRESSION

Neuroimaging has been a powerful tool to map actual changes in the brain structure of depressed patients that might be directly related to their symptoms of depression.^87^ Some imaging studies of brain structure have shown smaller hippocampal volume, with the chronicity of depression correlating to a reduction in hippocampal volume. Though the meaning of these findings is unclear, other studies have shown increased amygdala volume. Studies have found reductions in the volume of the frontal cortex, with some studies showing specific reductions in sub‐regions of the frontal cortex, including the orbitofrontal cortex.^87^ Findings of an increase in white matter lesions in elderly patients with depression have been replicated and correlated with late‐onset depression, as well as impairments in social and cognitive function. These findings point to alterations in a circuit of brain regions hypothesized to include the frontal cortex, hippocampus, amygdala, striatum, and thalamus, which underlie symptoms of depression.^87^, ^88^ These structural changes in depression affect connections between certain brain regions, or nuclei.

It has been shown that brain RAS affects these brain regions and nuclei differentially.^89^ A smaller hippocampus is associated with gene variants of the RAS. Genetic variants at three *AGTR1* SNPs (rs2638363, rs1492103, and rs2675511) were independently associated with accelerated hippocampal volume loss over the 4‐year follow‐up period in the right but not left hemisphere.^89^ Intriguingly, these *AGTR1* risk alleles also predicted worse episodic memory performance but were not related to other cognitive measures. Risk genetic variants of the RAS may accelerate memory decline in older adults, an effect that may be conferred by accelerated hippocampal volume loss.^89^ Molecules involved in this system may hold promise as early therapeutic targets for late‐life neuropsychiatric disorders. In addition, brain RAS regulates the functional and structural integrity of the amygdala.^90^

Furthermore, brain RAS integrates the connections among different brain nuclei and regions. AT1 receptors are primarily distributed in areas that control stress responses, such as the median eminence in the hypothalamic PVN, anterior pituitary gland, amygdala, septal nuclei, and hippocampus.^91^ Stress results in the formation of Ang II in the thalamus and other parts of the brain that contribute to catecholamine release.^91^ As AT1 and AT2 receptors are found in the HPA axis, HPA plays an important role in stress and stress‐related behavior.^92^ Activation of corticotropin‐releasing hormone (CRH) gene expression was found via AT1 receptor activation in immobilization‐induced stress. Treatment with ARB (telmisartan, candesartan, and valsartan) or ACE inhibitors reduces CRH‐induced adrenocorticotropic hormone (ACTH) and corticosterone release in spontaneously hypertensive rats. They also decreased pituitary sensitivity to CRH and reduced hypothalamic CRH expression, which ultimately led to a reduction in stress.^92^

Taken together, brain RAS regulates the functional activity and structural integrity of different brain regions involved in depression neuropathology. Different preclinical and clinical studies have highlighted the possible role of brain RAS and RAS modulators in the pathogenesis and clinical manifestations of depression.

### 5.1 Clinical findings

Different clinical studies indicated that over‐activity of brain RAS is linked with the development of mood and depressive symptoms.^93^, ^94^ The possible role of the RAS in hypertension and emotional disorders is well proven. Evidence points to the relationship between exaggerated RAS activity and depression and anxiety, partly through the induction of neuroinflammation, stress, and oxidative stress.^94^ Thus, blocking the RAS affords a theoretical basis for future treatment of anxiety and depression.^94^ Hertzman et al.^93^ revealed that ACEI lisinopril improves depressive symptoms in patients with MDD. Therefore, targeting brain RAS could reduce neuronal inflammation and oxidative stress.^94^ A case–control study that involved 972 diabetic patients with newly diagnosed depression and 972 diabetic patients without depression showed that the use of ACEIs were more effective than other antihypertensive drugs in the reduction of depressive symptoms.^95^ In addition, the use of calcium channel blockers and beta blockers in hypertensive patients increased the risk for the development of depression.^95^ Analysis of hypertensive patients from the hospital database with follow‐up for 5 years showed those on ACEIs or ARBs had a low risk for mood disorders.^96^ It has been shown that ACEIs such as enalopril and captopril reduce the negative emotional effects of hypertension.^97^ In particular, different studies highlighted that captopril has an antidepressant effect mainly in hypertensive patients.^98^ A previous clinical trial reported that ACEI captopril reduced depressive symptoms in hypertensive patients as compared to other hypertensive agents.^99^ Likewise, ACEI enalopril is more effective than beta blockers in ameliorating stressful events in hypertensive patients.^100^ Of interest, ACEI candesartan improves depressive symptoms in diabetic patients by regulating the HPA axis.^101^ A population‐based study revealed that hypertensive patients on ACEIs or ARBs showed less depressive symptoms compared to other antihypertensive agents.^102^ A meta‐analysis and systematic review confirmed that ACEIs and ARBs are more effective than other antihypertensive agents in reducing depressive and anxiety disorders in hypertensive patients.^103^ It has been shown that ACEIs and ARBs reduce the use of antidepressant drugs in hypertensive patients with depression.^104^ These clinical findings illustrated that ACEIs and ARBs are effective to reduce depressive symptoms in hypertensive patients with depression, though the underlying mechanism of their antidepressant effects was suggested to be mediated by anti‐inflammatory and antidepressant effects.

However, despite these findings, some studies proposed a link between prolonged use of ARBs and an increasing risk of suicide; nevertheless, the underlying mechanism was not fully elucidated.^72^, ^73^ Thus, preferential use of ACEIs instead of ARBs should be considered, especially in patients with mental disorders.^105^ Prolonged use of ARBs triggers a compensatory increase of brain AngII, leading to alteration of neurotransmitters and suicidal thought; nonetheless, this effect was not seen in ACEIs, which reduce AngII.^106^ Supporting this notion, *ACE* gene polymorphism, which increases AngII, is associated with suicide.^106^ In addition, higher AngII triggers the release of substance P, which affects the hypothalamic–pituitary–adrenal (HPA) axis, leading to stress and depressive symptoms.^107^ In addition, increasing circulating and central AngII in response to ARBs through activation of the AT1 receptor promotes inflammatory changes that induce mood and depressive disorders.^108^ These considerations suggest that ACEIs are safer than ARBs in hypertensive patients with a risk history of depression and other mood disorders.

It has been shown that RAS plays a critical role in the pathogenesis of depression.^26^ Furthermore, polymorphism and levels of ACE are increased in the Iranian population and increase the risk of depression.^109^ A case–control study that involved 191 patients with depression and 104 healthy controls showed that plasma ACE activity was higher in patients with depression as compared to the controls.^109^ Different studies highlighted that ACE polymorphisms affect antidepressant response, cognitive function, and suicidal behavior.^110^, ^111^ In relation to aldosterone, which is activated by AngII, it has been shown that patients with primary hyperaldosteronism experience depressive symptoms.^112^ As well, the high salivary concentration of aldosterone is correlated with depression severity in patients with MDD in a sex‐dependent manner.^113^ A cohort study on 60 patients with MDD (23 men and 37 women) showed that the salivary concentration of aldosterone was higher in women as compared to men.^113^ However, MDD patients with suicidal behavior have low plasma concentrations of aldosterone as compared with suicidal behavior without MDD.^114^ Besides, the aldosterone receptor antagonist spironolactone provokes depressive symptoms and reduces the efficacy of antidepressant drugs.^115^ These findings indicated a potential controversy regarding the role of aldosterone in depression neuropathology. In addition, ACEIs seem to be more appropriate for the alleviation of depressive symptoms in patients with depression.

### 5.2 Preclinical findings

Various preclinical studies have highlighted that RAS is augmented in depression. The AngII level and its expression are more intricate in the pathogenesis of depression. Targeting RAS either by gene deletion or by pharmacological inhibition leads to an antidepressant effect.^116^, ^117^ Deletion of the Ang gene reduces depressive‐like behavior in mice,^116^ and the use of ACEI captopril attenuates depression in rats.^117^ Many preclinical findings proposed that ARBs lead to antidepressant effects.^118^, ^119^ The AT1 receptor blocker irbesartan has an antidepressant effect in a depression mouse model.^118^ Similarly, the AT1 receptor blocker telmisartan reduces depression in diabetic rats. Besides, ACEIs such as candesartan produce an antidepressant effect in animal model study.^120^ The underlying mechanisms for the antidepressant effects of ARBs and ACEIs might be related to the upregulation of ACE2/Ang1‐7/MASR. Different experimental studies have observed that overexpression of ACE2 and Ang1‐7 in transgenic rats promotes antidepressant and antianxiety effects that are reversed by using MASR antagonists.^121^, ^122^ Supporting this notion, central administration of Ang1‐7 attenuates oxidative stress in the amygdala, and the use of MASR antagonists reverses the antidepressant effect of ACEI captopril in hypertensive rats.^123^, ^124^

The neuroprotective role of AngII/AT2 and AngII/Ang1‐7/MASR is related to their anti‐inflammatory and vasodilatory effects by inducing the release of nitric oxide (NO).^125^ The expression of AngII/AT2 and AngII/Ang1‐7/MASR in the brain is linked to the expression of AngII/AT1 and ACE.^126^ ACE2 reduces ACE function by reducing AngII levels; therefore, ACE2 has a dual effect, inhibiting AngII/AT1 and activating AngII/Ang1‐7/MASR in the brain.^127^ It has been shown that administration of ACE2 activator diminazen aceturate improves depressive and anxiety symptoms in mice by regulating HPA.^121^ MASR is involved in the regulation of emotional disorders, and the use of MASR antagonist A799 increases anxiety behavior in mice,^124^ and MASR‐deficient mice experience anxiety and depressive behaviors.^128^ Furthermore, the central effects of RAS inhibitors are also mediated through anti‐inflammatory and antioxidant effects. For example, in comparison with the antidepressant fluoxetine, the AT1 receptor blocker irbesartan reduces inflammatory and oxidative stress biomarkers as well as increases the 5HT level in chronic stress‐induced depression in mice.^117^ Likewise, ACEIs such as captopril, telmisartan, and candesartan attenuate central inflammation by inhibiting microglia, as evident by reducing inflammatory biomarkers such as tumor necrosis factor‐alpha (TNF‐α).^129^, ^130^ However, chronic captopril in mice provokes depressive‐like behavior by inhibiting regulatory T cells and induction of AngII‐mediated oxidative stress.^131^

Collectively, these verdicts indicated that depression neuropathology is linked with RAS over‐activity, and selective inhibition of AngII/AT1 or activation of AngII/AT2 and AngII/Ang1‐7/MASR could be a therapeutic way for attenuating depression.

## 6 MECHANISTIC ROLE OF BRAIN RAS IN DEPRESSION

### 6.1 RAS and 5HT

According to the monoamine theory, 5HT is the main neurotransmitter that is reduced in depression.^132^ The 5HT theory was based on the depressogenic activity of reserpine, which depletes 5HT and other monoamines in the presynaptic terminals, and the effects of antidepressants, which increase 5HT in the synaptic cleft.^133^ A case–control study showed that subjects with suicidal attempts have low serum 5HT levels. Treatment with selective serotonin reuptake inhibitors (SSRIs) improves depressive symptoms but not serum 5HT levels.^134^ A systematic review and meta‐analysis illustrated that brain 5HT is not associated with depression, and long‐term therapy with antidepressants reduced rather than increased 5HT.^135^ Even so, 5HT is still implicated in the pathogenesis of depression.

It has been revealed that AngII can inhibit the synthesis and release of 5HT in the hippocampus.^136^ In addition, AngII increases the turnover and metabolism of 5HT, as evidenced by an increase in its metabolite 5HIAA.^136^ ARBs have antidepressant effects through modulation of 5HT metabolism.^136^ Remarkably, AngII at a higher concentration promotes 5HT biosynthesis, though at a lower concentration, AngII inhibits 5HT biosynthesis.^137^ However, AngII is negatively associated with 5HT biosynthesis. Back to this concept, many experimental studies confirmed that ARBs and ACEIs increase 5HT in the hippocampus and prefrontal cortex.^138^, ^139^, ^140^ Furthermore, the neuroprotective Ang1‐7, which antagonizes AngII, improves brain 5HT and depressive symptoms in transgenic rats with low angiotensinogen levels.^141^ Klempin et al.^142^ showed that depletion of brain ACE2 reduces brain 5HT, leading to significant impairment of neurogenic response in mice. Of note, intestinal ACE2 enhances the absorption of tryptophan, which is a precursor of 5HT.^143^ ARBs and ACEIs, via activation of the ACE2/Ang1‐7/MASR axis, improve neurogenesis and 5HT biosynthesis.^143^ Therefore, dysregulated brain RAS is linked with the development and progression of depression by inhibiting 5HT. Thus, inhibition of the classical RAS pathway by ARBs and ACEIs mitigates brain 5HT biosynthesis and reduces depressive symptoms. As well, ACE2 activators or enhancement of non‐classical RAS pathways by ARBs and ACEIs also improve brain 5HT synthesis and release.

### 6.2 RAS and BDNF

BDNF is a member of the neurotrophins protein family that is concerned with neuronal injury resistance.^144^ BDNF acts mainly on tyrosine kinase receptor B (TrkB) and, to a lesser extent, on the p75NT receptor (p75NTR).^144^ BDNF is released from peripheral tissues and the CNS, mostly the hypothalamus, hippocampus, and limbic system.^145^ It has been reported that the deregulation of BDNF is linked to the pathophysiology of depression and other mood disorders.^36^ BDNF/TrkB signaling is essential for hippocampal long‐term potentiation (LTP) via activation of extracellular signal‐regulated protein kinase (ERK) and mitogen‐activated protein kinase (MAPK).^146^ For example, increasing the activity of HPA and reducing estradiol cause dysfunction of the BDNF signaling pathway, which leads to depression.^147^

Different preclinical studies established that BDNF expression in the hippocampus and cerebral cortex was reduced in animal model studies.^148^ Decrease of BDNF and other neurotrophic factors in the prefrontal cortex and hippocampus and stimulation of neurotrophic factors, which is mediated by antidepressant agents, can attenuate neuronal atrophy.^148^ Likewise, chronic administration of the antidepressant duloxetine in rats for 21 days improves BDNF levels.^149^ In addition, acute stress episodes in rats treated with antidepressants activate increased BDNF levels.^149^ Interestingly, reduction of BDNF expression in specific forebrain regions provokes the development of depression, and upregulation of BDNF expression in these areas is mediated by the action of antidepressant agents.^150^ In addition, antidepressant effects mediated by BDNF are differential according to specific brain areas as well as in healthy and depressed animals.^45^ Deletion of the BDNF gene in forebrain regions leads to hyperactivity in male mice, but depressive‐like behavior in female mice,^150^ suggesting a sex‐dependent effect of BDNF.

Moreover, numerous clinical studies have exposed that BDNF levels are condensed in patients with depression.^151^, ^152^, ^153^, ^154^ A postmortem brain analysis of 21 MDD patients and 21 healthy controls showed that BDNF expression in the amygdala was abridged in MDD patients compared to healthy controls.^154^ BDNF gene expression and BDNF/TrkB signaling in the anterior cingulate cortex are found in the brains of 51 postmortem patients with MDD compared to 102 healthy controls.^154^ Additionally, low BDNF expression is correlated with depression severity.^155^ A meta‐analysis that included 11 studies showed that BDNF serum level was reduced in patients with depression, and prolonged use of antidepressant drugs was linked with an increase in BDNF serum level.^155^ A recent study observed that training exercise improves depressive symptoms in patients with depression by increasing circulating BDNF serum levels.^156^ However, pro‐BDNF, which acts on p75NTR, is augmented in depression.^157^ A case–control study on 42 patients with depression and 40 healthy controls revealed that pro‐BDNF and BDNF serum levels were augmented and decreased correspondingly in patients with depression^157^ signifying an interruption in the conversion of pro‐BDNF to BDNF. These findings indicated that the BDNF/TrkB axis is extremely downregulated in patients with depression.

Importantly, RAS regulates the expression of the BDNF/TrkB axis in different ways.^158^ For example, exaggerated RAS in experimental diabetic rats induces downregulation of the BDNF/TrkB axis, leading to depression. Inhibition of overactive RAS by AT1 receptor antagonist losartan restores BDNF/TrkB activity in astrocytes.^158^ Furthermore, it has been observed that AngII reduced BDNF expression by inducing the expression of toll‐like receptor 4 (TLR4) and pro‐inflammatory NF‐κB.^159^ Administration of a small non‐antihypertensive dose of AT1 receptor blocker candesartan (0.1 mg/kg) in mice for 15 days inhibits LPS‐induced memory impairment by increasing expression of the BDNF/TrkB axis, which inhibits TLR4 and NF‐κB.^159^ Likewise, administration of the AT1 receptor antagonist valsartan promotes neurogenesis via a BDNF‐dependent pathway in mice.^118^ Remarkably, valsartan triggers antidepressant effects in mice in a dose‐dependent manner.^118^ It has been documented that inhibition of the central ACE and AT1 receptors by captopril and valsartan, respectively, improved cognitive function in rats with experimental AD by increasing BDNF expression.^160^ As well, numerous experimental studies showed that activation of the Ang1‐7/MASR axis promotes expression of the BDNF/TrkB axis, thereby reducing cognitive dysfunction and memory impairment.^161^, ^162^ Notoriously, AngIV has a neuroprotective effect against memory impairment in diabetic rats by increasing the expression of neuronal BDNF.^163^ Peripheral administration of AngIV reduces spatial memory impairments by inhibiting hippocampal oxidative stress and activating hippocampal BDNF.^163^

**FIGURE 3:**
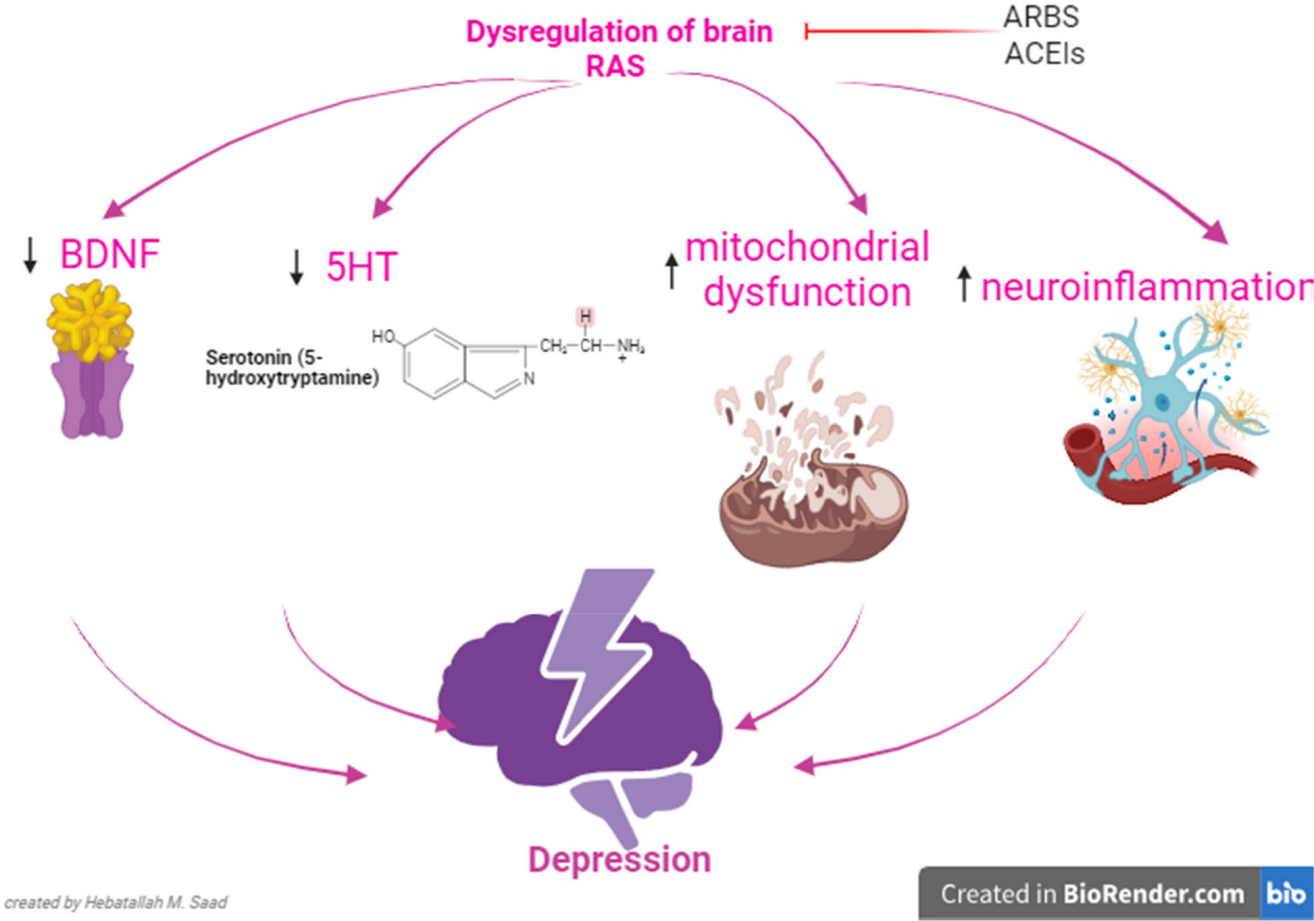
Role of serotonin (5HT) in depression. Dysregulation of brain renin–angiotensin system (RAS) triggers the development and progression of depression through the reduction of brain 5HT and expression of BDNF and the induction of mitochondrial dysfunction, oxidative stress, and neuroinflammation. Therefore, inhibition of central classical RAS by angiotensin‐ACEIs and ARBs and activation of non‐classical RAS prevent the development of depression by regulating 5HT, BDNF, mitochondrial dysfunction, oxidative stress, and neuroinflammation.

These observations propose that dysregulation of central RAS may increase depression neuropathology by reducing the expression and functional activity of the BDNF/TrkB axis. Targeting of central RAS through inhibition of the AngII/AT1 pathway by ACEIs and ARBs or by activating AngII/AT2 and AngII/Ang1‐7/MASR by AT2 and MASR activators may attenuate depression by increasing BDNF (Figure 3).

### 6.3 RAS, mitochondrial dysfunction and oxidative stress

It has been shown that ROS are produced constantly by all body tissues that are removed by endogenous antioxidant capacity.^164^ When there is an imbalance between ROS generation and antioxidant capacity, oxidative stress is developed.^165^ The mitochondria are the major site for the generation of ROS, which affect mitochondrial DNA, leading to more ROS generation.^166^ Notably, the brain is extremely vulnerable to the effects of oxidative stress due to advanced metabolic activity, the generation of ROS, and its lower antioxidant capacity.^167^ Chronic stressful condition in depression encourages oxidative stress and mitochondrial dysfunction.^168^ Depression‐induced oxidative stress is involved in the development of structural and functional changes, including the reduction of the size of the hippocampus and frontal cortex and the dysfunction of synaptic plasticity.^168^ Functional brain changes in depression are suggested to develop according to the oxidative stress hypothesis of depressive disorders.^168^, ^169^ Oxidative stress in mood disorders, including depression, may extend to the periphery, causing distant organ damage. A nested case–control study observed that peripheral biomarkers of oxidative stress were higher in patients with mood disorders compared to controls.^167^ Pro‐oxidants and oxidants are advanced in depression, causing an alteration in the permeability of the BBB and the development of systemic oxidative complications.^170^ Treatment with antioxidant and antidepressant agents overturned depressive symptoms in experimental diabetes.^171^, ^172^ Higher brain oxidative stress depletes 5HT, which has antioxidant effects, leading to the progression of depression.^173^, ^174^ Of interest, antidepressant drugs like fluoxetine and mirtazapine, which inhibit the reuptake of 5HT reduce the severity of oxidative stress.^175^, ^176^ Thus, depletion of 5HT exacerbates depressive symptoms through the induction of oxidative stress.^176^

On the other hand, over‐activation of RAS is associated with the development and progression of mitochondrial dysfunction and oxidative stress.^177^ Dysregulation of RAS and associated mitochondrial dysfunction and oxidative stress might be a possible link between obesity and insulin resistance.^177^ Unbalanced and activated AngII can cause direct mitochondrial dysfunction and oxidative stress in the skeletal muscles of experimental mice.^178^ It has been reported that AngII‐induced vascular injury is mediated through the induction of mitochondrial dysfunction and oxidative stress.^179^ Similarly, exaggerated AngII and linked mitochondrial dysfunction and oxidative stress are associated with cognitive impairment and the development of neurodegenerative diseases.^180^ However, Ang1‐7 reverses AngII‐induced cerebral endothelial dysfunction by inhibiting NADPH oxidase and the generation of ROS.^181^ Therefore, inhibition of AngII/AT1 and activation of AngII/AT2 and Ang1‐7/MASR axes may reduce brain mitochondrial dysfunction and oxidative stress. Ketan et al.^182^ proposed that ARBs attenuate brain mitochondrial dysfunction and oxidative stress. A preclinical study conducted by Gupta et al.^183^ demonstrated that ARB azilsartan attenuates experimental stroke in rats by inhibiting mitochondrial dysfunction, oxidative stress, and associated neuroinflammation. Notoriously, AngII inhibits transcription of the *ACE2* gene, leading to a lowering of ACE2 expression. Therefore, ACEIs and ARBs increase the expression of ACE2 and promote the Ang1‐7/MASR axis, which has a neuroprotective effect.^184^, ^185^

These findings revealed that dysregulated AngII, linked mitochondrial dysfunction, and oxidative stress may be involved in the pathogenesis of depression. Inhibition of brain RAS by ACEIs and ARBs not only mitigates AngII‐induced neuronal mitochondrial dysfunction and oxidative stress through suppression of AngII/AT1 but also increases the ACE2/Ang1‐7/MASR axis.

### 6.4 RAS and neuroinflammation

Neuroinflammation is an immune response of the CNS to exogenous infectious agents or endogenous neurological disorders, as in neurodegenerative diseases.^186^ Microglia and astrocytes are involved in the development of neuroinflammation; nonetheless, peripheral immune cells that traverse injured BBB can involve the development of neuroinflammation in chronic inflammatory disorders.^165^ Neuroinflammation in the acute phase is defended to eradicate the underlying cause, though chronic neuroinflammation may induce neuronal injury, synaptic dysfunction, and exacerbation of brain neuropathology. It has been shown that neuroinflammation affects synaptic plasticity, inhibits hippocampal neurogenesis, and dysregulates HPA.^187^ As well, chronic stress triggers microglial activation and the release of pro‐inflammatory cytokines, which inhibit neurogenesis and induce neurodegeneration.^187^ Of note, depression is associated with low‐grade inflammatory reactions due to the activation of microglia and the release of pro‐inflammatory cytokines.^188^ Besides, exaggerated inflammatory reactions are linked with the progression of depression.^189^ A higher level of CRP is regarded as a potential biomarker of depression severity.^190^ A clinical study observed that inflammatory biomarkers were increased in both plasma and CSF in patients with depression.^191^ Therefore, inhibitors of pro‐inflammatory cytokines such as etanercept have antidepressant effects.^192^ Over‐activity of brain RAS is associated with the development and progression of neuroinflammation.^193^ The classical RAS pathway (AngII/AT1) activates T cells and macrophages, leading to the release of pro‐inflammatory cytokines and the development of neuroinflammation. However, the non‐classical RAS pathway (ACE2/Ang1‐7/MASR axis) counteracts the activating effect of AngII/AT1 on the immune cells and the progression of neuroinflammation.^193^ Impairment of BBB facilitates the entry of peripheral AngII into the CNS that normally does not pass.^193^ Central AngII triggers the activation of brain NADPH oxidase, leading to the generation of ROS and the development of oxidative stress, which promotes the progression of neuroinflammation.^194^ Targeting neuroinflammation by inhibiting RAS is an essential step, mainly in patients with resistance depression.^195^ It has been reported that ACEIs and ARBs can attenuate neuroinflammation by blocking the expression of pro‐inflammatory cytokines.^196^ An experiment showed that ARB losartan attenuates LPS‐induced neuroinflammation in mice through suppression of the expression of pro‐inflammatory cytokines.^196^ Likewise, ACEI candesartan tempers LPS‐induced neuroinflammation by inhibiting microglial activation and expression of pro‐inflammatory cytokines and regulating neuronal insulin signaling in rats.^197^ Interestingly, candesartan at a low dose increases the expression of the anti‐inflammatory cytokines IL‐10 and AT2.^197^ Therefore, ARBS and ACEIs seem to have neuroprotective and antidepressant effects by attenuating neuroinflammation. Supporting this idea, experimental studies revealed that ARBS and ACEIs have antidepressant effects.^117^, ^198^ Losartan and irbesartan inhibit depressive‐like behavior in mice.^117^, ^198^ In addition, losartan and telmisartan attenuate diabetes‐induced depression in experimental animals.^119^, ^158^ Furthermore, ACEI captopril mitigates depressive disorders in experimental rats by inhibiting hypothalamic microglia.^199^

These observations indicated that inhibition of the classical RAS pathway can reduce depression through attenuation of neuroinflammation.

## 7 DEPRESSION THERAPEUTIC MODALITIES AND BRAIN RAS

Antidepressant drugs are one of the most commonly used in the management of depression.^200^ SSRIs are frequently used in the management of depression compared to tricyclic antidepressants (TCAs) due to their low incidence of adverse effects.^200^, ^201^ Previous preclinical studies indicated that TCAs had the ability to reduce the function activity of brain AngII.^202^, ^203^ Genetic polymorphisms in ACE affect the response to antidepressant agents.^202^ A clinical trial on patients with depression showed that the G8790A genetic variant of ACE2 is linked with a better response to the efficacy of SSRIs.^204^ It has been shown that the action of AngII is reduced by antidepressants, and this signifies the complex interplay of different mechanisms involved in response to therapy. Furthermore, TCA imipramine can attenuate AngII‐induced depressive‐like behaviors by inhibiting hippocampal microglial activation and HPA axis hyperactivation in mice.^205^ A cohort study on 200 newly diagnosed depressed patients treated with fluoxetine or sertraline for 12 weeks showed that genetic variants of RAS may influence or be an indicator for a better response to sertraline but not fluoxetine,^206^ suggesting a drug‐specific effect on brain RAS. These findings indicated that antidepressant agents may induce antidepressant effects through modulation of brain RAS.

In addition, psychotherapy is also involved in the management of depression. Patients in regional, rural, and remote communities experience perennial difficulties accessing mental health treatments in a timely manner, which contributes to inequitable outcomes.^207^ However, the effect of psychotherapy on brain RAS is still not identified. In the treatment of depression, when pharmacotherapy, psychotherapy, and the oldest brain stimulation techniques are deadlocked, the emergence of new therapies is a necessary development. The field of neuromodulation is very broad and controversial for treatment‐resistant depression. Neuromodulatory techniques including magnetic seizure therapy; focal electrically administered seizure therapy, transcranial pulsed electromagnetic fields, transcranial direct current stimulation, epidural cortical stimulation, trigeminal nerve stimulation, transcutaneous vagus nerve stimulation, transcranial focused ultrasound, near‐infrared transcranial radiation, and closed‐loop stimulation could be effective in the management of depression.^208^, ^209^ Notably, transcranial direct current stimulation increased parasympathetic activity and decreased sympathetic activity by modulating brain RAS, suggesting the importance of this neuromodulatory technique in the management of stress‐related disorders and depression.^210^ The underlying mechanisms of transcranial direct current stimulation in depression are related to the regulation of resting membrane potential, spontaneous neuronal firing rates, synaptic strength, cerebral blood flow, and neuronal metabolism.^210^ However, the effects of neuromodulatory techniques on brain RAS are not fully elucidated.

Taken together, dysregulation of brain RAS triggers the development and progression of depression through the reduction of brain 5HT and expression of BDNF and the induction of mitochondrial dysfunction, oxidative stress, and neuroinflammation. Therefore, inhibition of central classical RAS by ARBS and ACEIs and activation of non‐classical RAS prevent the development of depression by regulating 5HT, BDNF, mitochondrial dysfunction, oxidative stress, and neuroinflammation (Figure 3).

## 8 CONCLUSIONS

Depression is a neuropsychiatric disorder characterized by abnormal thoughts and suicidal attempts. The pathophysiology of depression is related to the deficiency of 5HT, which is derived from Trp. Mitochondrial dysfunction, oxidative stress, and neuroinflammation are intricate in the pathogenesis of depression. Particularly, RAS is involved in the pathogenesis of depression, and all components of RAS have been recognized in various brain regions, and the AngII level was higher in the brain than in the peripheral circulation. Brain RAS has two arms: the harmful one is mediated by the AngII/AT1 receptor, and the protective arm is mediated by the AngII/AT2 receptor and the ACE2/Ang1‐7/MASR axis. Depression increases the risk of hypertension and increases the prevalence of depression among hypertensive patients, suggesting a potential association between them. Therefore, early management of depression and hypertension prevents the development and progression of a pathogenic link between them. One of the most commonly used antihypertensive agents are ARBs and ACEIs, which modulate peripheral and central RAS. Different findings revealed that ACEIs and ARBs may be effective in treating depression. Therefore, brain RAS is implicated in the pathogenesis of depression, and modulation of this system may ameliorate the development and progression of depression. Depression neuropathology is linked with RAS over‐activity, and selective inhibition of AngII/AT1 or activation of AngII/AT2 and AngII/Ang1‐7/MASR could be a therapeutic way of attenuating depression. Overall, dysregulation of brain RAS triggers the development and progression of depression through reduction of brain 5HT and expression of BDNF and induction of mitochondrial dysfunction, oxidative stress, and neuroinflammation. Therefore, inhibition of central classical RAS by ARBS and ACEIs and activation of non‐classical RAS prevent the development of depression by regulating 5HT, BDNF, mitochondrial dysfunction, oxidative stress, and neuroinflammation. This review suggests preclinical and clinical studies in this regard.

## AUTHOR CONTRIBUTIONS

Naif H. Ali, Hayder M. Al‐Kuraishy, Ali I. Al‐Gareeb, and Ali K. Albuhadily conceptualized the manuscript, wrote, edited, and reviewed the main text, and approved the final edition of the manuscript. Rabab S. Hamad, Athanasios Alexiou, Marios Papadakis, Hebatallah M. Saad, and Gaber El‐Saber Batiha prepared the figures, wrote, corrected, amended, and approved the final edition of the manuscript.

## FUNDING INFORMATION

Open Access funding enabled and organized by Projekt DEAL. This work was supported by the University of Witten‐Herdecke Germany.

## CONFLICT OF INTEREST STATEMENT

The authors declare no conflict of interest.

## Data Availability

Data sharing are not applicable to this article as no data sets were generated or analyzed during the current study.
