# Correction to “Role of brain renin–angiotensin system in depression: A new perspective”

**DOI:** 10.1111/cns.14884

**Published:** 2024-07-23

Ali NH, Al‐Kuraishy HM, Al‐Gareeb AI, et al. Role of brain renin–angiotensin system in depression: A new perspective. *CNS Neurosci Ther*. 2024;30:e14525. doi:10.1111/cns.14525

In Introduction section, the sentence “Depression affects 3.5% of the general population.^2^” is incorrect. The sentence should have read: “Approximately 280 million people globally suffer from depression, representing about 3.8% of the world's population.^2,211^”, where Reference 211 is: WHO Fact sheets for Depressive disorder (depression) Retrieved from https://www.who.int/news‐room/fact‐sheets/detail/depression.

Additionally, in Introduction section, in the sentence “The risk factors for development are multifactorial, including stressful life events,^7^ borderline personality disorders,^8^ chronic use of sedatives and hypnotics, and adverse effects from long‐term use of β‐blockers, antipsychotic drugs, isotretinoin, and antimigraine medications.^9,10^”, we would like to add another reference and the sentence should have read: “The risk factors for development are multifactorial, including stressful life events,^7^ borderline personality disorders,^8,212^ chronic use of sedatives and hypnotics, and adverse effects from long‐term use of β‐blockers, antipsychotic drugs, isotretinoin, and antimigraine medications.^9,10^”, where reference 212 is: National Institute of Mental Health. (2024, April). Borderline Personality Disorder. U.S. Department of Health and Human Services, National Institutes of Health. Retrieved May 13, 2024, from https://www.nimh.nih.gov/health/topics/borderline‐personality‐disorder.

Also, in Introduction section, in the sentence “Moreover, psychiatric disorders such as bipolar disorders, major depressive disorder (MDD) episodes, and seasonally affected disorders increase the risk of the development of depression.^11^”, we would like to add another reference and the sentence should have read: “Moreover, psychiatric disorders such as bipolar disorders, major depressive disorder (MDD) episodes, and seasonally affected disorders increase the risk of the development of depression.^11,213^”, where reference 213 is: Øverland S, Woicik W, Sikora L, et al. Seasonality and symptoms of depression: A systematic review of the literature. *Epidemiol Psychiatr Sci*. 2020;29:e31. doi:10.1017/S2045796019000209.

In Introduction section, in the sentence “Additionally non‐psychiatric disorders, including nutritional deficiencies,^12^ infectious diseases,^13^ Addison disease,^14^ hypothyroidism,^15^ Cushing disease,^16^ hyperparathyroidism,^17^ Parkinson's disease (PD)^18^ and multiple sclerosis,^19^ trigger the incidence of depression.”, we would like to replace reference 13 as follows: Yuan, K., Zheng, YB., Wang, YJ, et al. A systematic review and meta‐analysis on prevalence of and risk factors associated with depression, anxiety and insomnia in infectious diseases, including COVID‐19: a call to action. *Mol Psychiatry*. 2022;27:3214‐3222. https://doi.org/10.1038/s41380‐022‐01638‐z.

We apologize for this error.

